# Editorial: Reviews in neuropsychology

**DOI:** 10.3389/fpsyg.2023.1327860

**Published:** 2023-11-10

**Authors:** Yang Zhou, Xiansong Xia, Kai Yuan, Dongdong Qin

**Affiliations:** ^1^Key Laboratory of Traditional Chinese Medicine for Prevention and Treatment of Neuropsychiatric Diseases, Yunnan University of Chinese Medicine, Kunming, Yunnan, China; ^2^The First School of Clinical Medicine, Yunnan University of Chinese Medicine, Kunming, Yunnan, China; ^3^Open and Shared Public Science and Technology Service Platform of Traditional Chinese Medicine Science and Technology Resources in Yunnan, Yunnan University of Chinese Medicine, Kunming, Yunnan, China; ^4^The Department of Educational Administration, Yunnan University of Chinese Medicine, Kunming, Yunnan, China; ^5^The Second School of Clinical Medicine, Yunnan University of Chinese Medicine, Kunming, Yunnan, China

**Keywords:** reviews, neuropsychology, neuroscience, psychology, brain functioning

Neuropsychology integrates the knowledge base of neuroscience and psychology, which can be widely used in preclinical and clinical fields to deeply explore the mechanisms of diseases related to physical and mental health disorders. The aim of this Research Topic “*Reviews in Neuropsychology*,” published in Frontiers in Psychology, was to highlight recent advances in neuropsychology and provide important directions and new possibilities for future studies. With this call for authors, we wanted to explore the association between cognitive behavioral manifestations in healthy or pathological subjects and the brain functioning through clinical and observational tools or electrophysiology and neuroimaging. This topic collected four contributions consisting of three systematic reviews and one systematic review and meta-analysis.

Emotion recognition and processing are thought to help build social interactions (Morese and Palermo, 2022). Research suggests that emotion recognition in older adults is more susceptible to disorders involving brain structures (Amlerova et al., 2022). Morellini et al. presented a systematic review to investigate and update the state of the literature in this important but undervalued field of “emotion recognition and processing in mild cognitive impairment (MCI) populations” over the past 10 years. It is worth noting that MCI individuals have emotion-specific deficits in emotion recognition. Although these results cannot be applied to the entire MCI population, they can predict the type of cognitive impairment associated with neurodegenerative diseases and help clinicians develop effective therapeutic strategies.

Sustained exposure to epileptogenic neurodevelopmental disorder, such as autism spectrum disorder (ASD), may impair language expression and communication cognitive skills (Braakman et al., 2012; Berg, 2016). Therefore, it is reasonable to recognize that seizures impair the language expression and communication cognitive abilities of children with ASD. Through systematically reviewing the published literature, Cano-Villagrasa et al. explored the effects of epilepsy on the development of cognitive and language skills in children with ASD. The results showed that epilepsy significantly damaged the cognitive and language abilities of ASD individuals and exerted a significant impact on the quality of daily life and basic activities.

From the perspectives of activity theory and neuroscience, Molina et al. used the PRISMA and PICOS guidelines to analyze current research trends on cognition, integrative complexity (IC), and decision-making. This systematic literature review included 31 papers, 19 of which were related to neuroscience, and the correlation between IC and decision-making outcomes was investigated in various situations. The results showed that the definition and classification of IC need to be precise and concrete to assess its relations with cognition and decision-making. Neuroscience methods show tremendous potential in unraveling the cognitive nature of IC.

Research on the effects of substance abuse and addiction on cognitive domains appears to have received insufficient attention (Kloft et al., 2021). In the systematic review and meta-analysis by Caetano et al. correlations regarding substance abuse and false memory formation were analyzed. The results demonstrated that individuals with a history of substance abuse had greater susceptibility to related and unrelated misrecognition/recall, but no effects on intrusion misrecognition/recall of critical lures were observed. The authors call for continued focus on (1) exploring the presence of polydrug use in false memory formation and (2) considering different types of false memories and their potential associations with relevant clinical variables.

Overall, the contributions in this special topic highlight the intricate nature of the challenges that the field of neuropsychology is currently encountering. We expect that this Research Topic will inspire more debates within the neuropsychological community, which can be translated into best practice applications in preclinical and clinical, public health, and policy settings.

## Author contributions

YZ: Writing—original draft, Writing—review & editing. XX: Writing—review & editing. KY: Writing—review & editing. DQ: Conceptualization, Supervision, Validation, Writing—review & editing.
